# The Mesenteric Masquerader: A Rare Primary Neuroendocrine Tumor of the Mesentery

**DOI:** 10.7759/cureus.113235

**Published:** 2026-07-23

**Authors:** Manogya Khanna, Subhash Chawla, Harsh Gupta, Mrugen Thakor, Vishesh Dhawan

**Affiliations:** 1 General Surgery, Maharishi Markandeshwar (Deemed to be University), Mullana, Ambala, IND; 2 Orthopaedics, Maharishi Markandeshwar Institute of Medical Sciences, Maharishi Markandeshwar (Deemed to be University), Mullana, Ambala, IND; 3 Pathology, Maharishi Markandeshwar Institute of Medical Sciences, Maharishi Markandeshwar (Deemed to be University), Mullana, Ambala, IND

**Keywords:** chromogranin, laparoscopy, mesentery, neuroendocrine tumor, primary mesenteric net, synaptophysin, well-differentiated net, who grade i

## Abstract

Primary neuroendocrine tumors (NETs) arising exclusively within the mesentery, without an identifiable gastrointestinal or pancreatic primary, are exceedingly rare and often present with non-specific symptoms, making preoperative diagnosis challenging. We report the case of an 80-year-old female who presented with a six-month history of right-sided abdominal pain and a palpable abdominal mass. Contrast-enhanced computed tomography of the abdomen revealed a well-defined soft tissue lesion measuring 7.1 × 4.4 × 5.3 cm in the right iliac fossa mesentery, with vascular supply from the superior mesenteric artery. The mass was successfully excised laparoscopically en masse. Histopathological examination demonstrated a WHO Grade I well-differentiated NET with lymphovascular invasion and negative resection margins. Immunohistochemistry showed positivity for pan-cytokeratin, synaptophysin, chromogranin, INSM1, and CD56. This case highlights the diagnostic challenge of primary mesenteric NETs and supports surgical resection as the definitive management. Careful postoperative surveillance is warranted due to the potential for recurrence.

## Introduction

Neuroendocrine tumors (NETs) are a heterogeneous group of neoplasms arising from the diffuse neuroendocrine cell system and most commonly originate from the gastrointestinal tract, pancreas, and bronchopulmonary system [1]. Primary NETs arising exclusively within the mesentery, without any identifiable gastrointestinal or pancreatic primary lesion, represent an exceptionally rare clinical entity, with only a limited number of cases documented in the literature [2,3].

Their non-specific clinical presentation - typically an asymptomatic or mildly symptomatic abdominal mass - and rarity frequently lead to diagnostic delay and misdiagnosis as other mesenteric tumors such as gastrointestinal stromal tumors (GISTs), desmoid tumors, or lymphomas [2]. We present a case of a primary mesenteric NET in an elderly female, highlighting the diagnostic workup, surgical management, and pertinent histopathological and immunohistochemical findings. This case report adheres to the CARE guidelines for case report reporting.

## Case presentation

An 80-year-old female was admitted with a six-month history of right-sided abdominal pain, moderate in intensity, with no relieving factors, aggravated on bending over and decreased bowel habits. Physical examination revealed a vague abdominal lump in the right iliac fossa (RIF) extending towards the umbilicus, measuring approximately 7 × 5 cm. The mass had a smooth surface, ill-defined margins, was non-tender, firm in consistency, did not move with respiration, and had restricted mobility.

**Figure 1:**
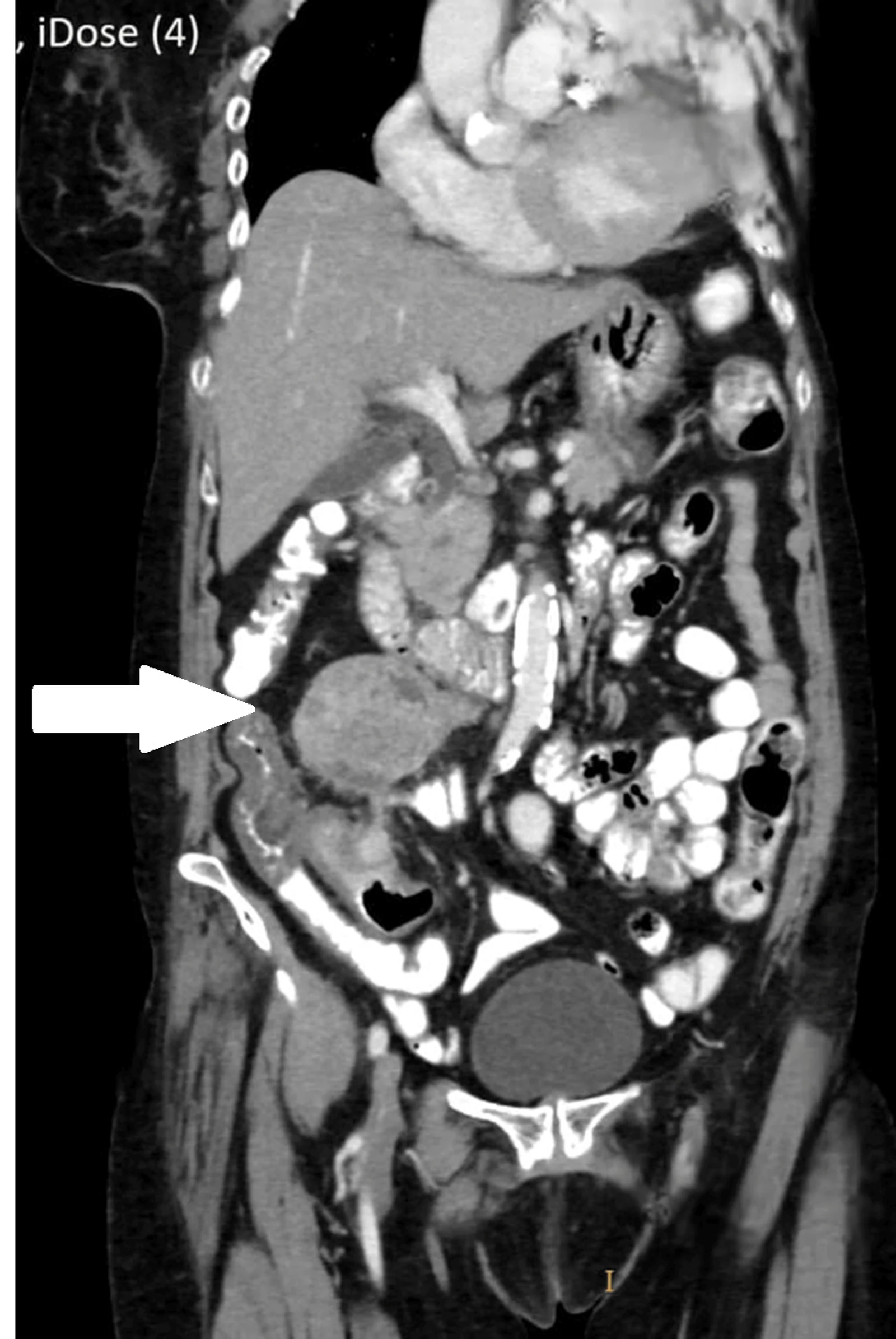
Contrast-enhanced computed tomography (CECT) Abdomen (coronal view) Demonstrating a well-defined soft tissue mesenteric mass (7.1x4.4x5.3 cm) in the right iliac fossa with vascular supply from the superior mesenteric artery.

**Figure 2:**
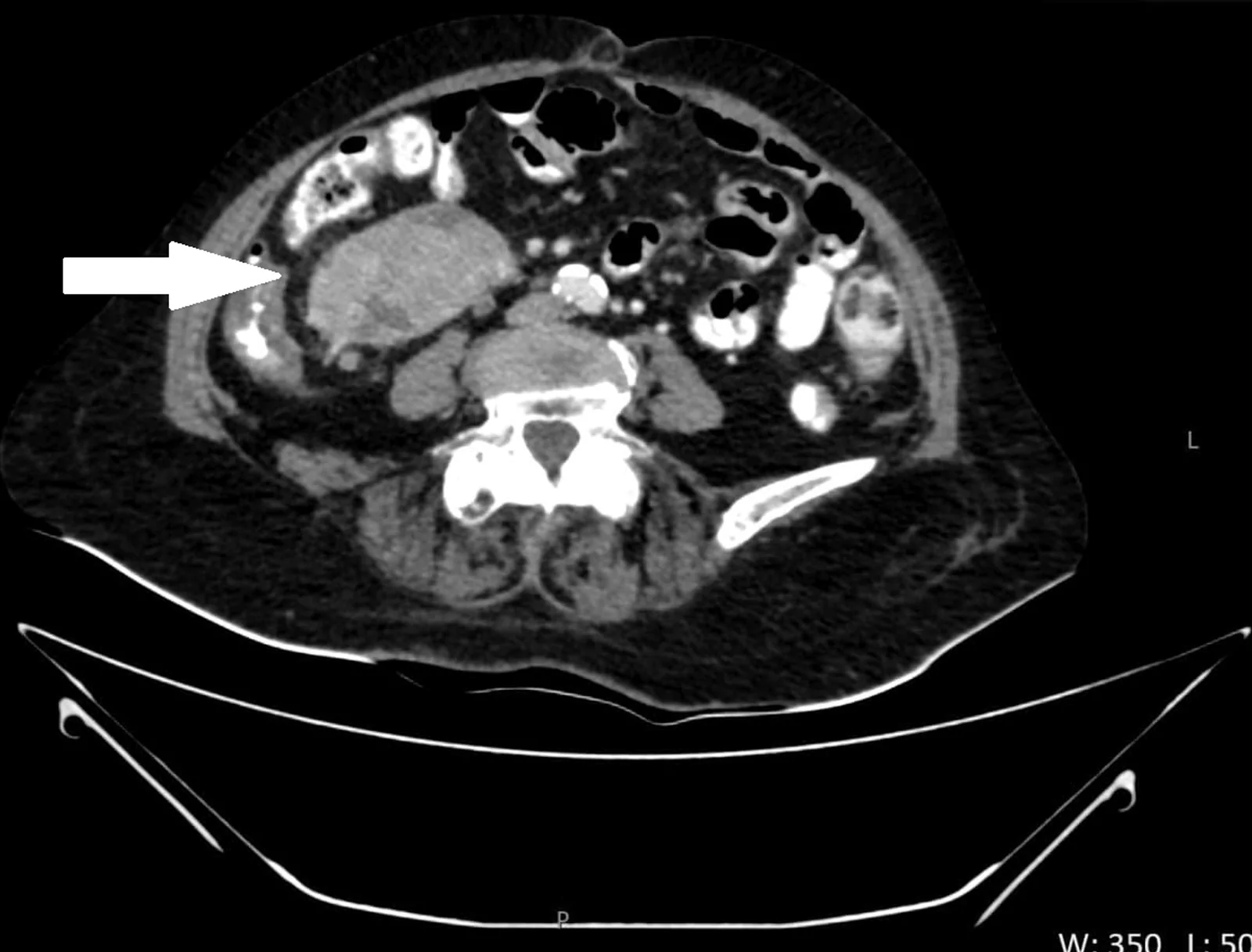
Contrast-enhanced computed tomography (CECT) Abdomen (axial view) Showing mesenteric mass in right iliac fossa.

Contrast-enhanced computed tomography (CECT) of the whole abdomen demonstrated a well-defined, circumscribed soft tissue density mass lesion in the RIF within the mesentery, measuring 7.1 × 4.4 × 5.3 cm. The mass received vascular supply from branches of the superior mesenteric artery (SMA). A stalk was identified extending from the mesenteric mass to the terminal ileum. No regional lymph node involvement was identified (Figures 1, 2). CT imaging showed a heterogeneous enhancement on post-contrast study, suggesting a hypervascular tumor. 

**Figure 3:**
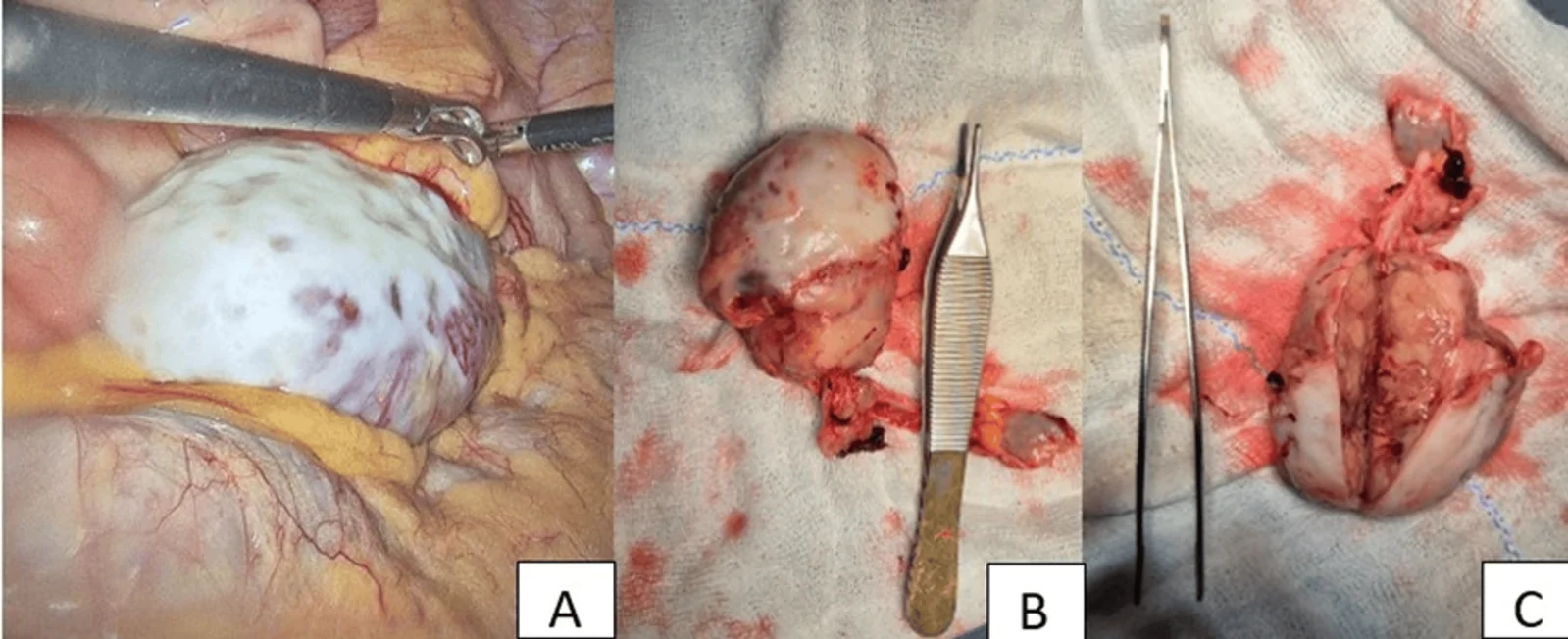
Intraoperative Images Intraoperative gross images of the mesenteric neuroendocrine tumor showing the laparoscopic appearance before excision (A), the intact excised specimen (B), and the cut section of the specimen (C).

The patient underwent diagnostic laparoscopy, which identified a vascular mass of approximately 7 × 5 cm adherent to the ileal mesentery, located approximately 80 cm proximal to the ileocaecal junction. Thorough exploration of the abdominal cavity revealed no evidence of additional masses or lesions involving the small or large intestine, stomach, liver, pancreas, or intraperitoneal rectum. The tumor was laparoscopically excised en masse, with ligation of all adhesions and the supplying vasculature (Figure 3).

The postoperative course was complicated by severe hypertension refractory to oral and intravenous antihypertensive medications, necessitating intravenous nitroglycerin (glyceryl trinitrate) infusion. Blood pressure normalized within 72 hours and the patient was discharged following satisfactory postoperative recovery.

**Figure 4:**
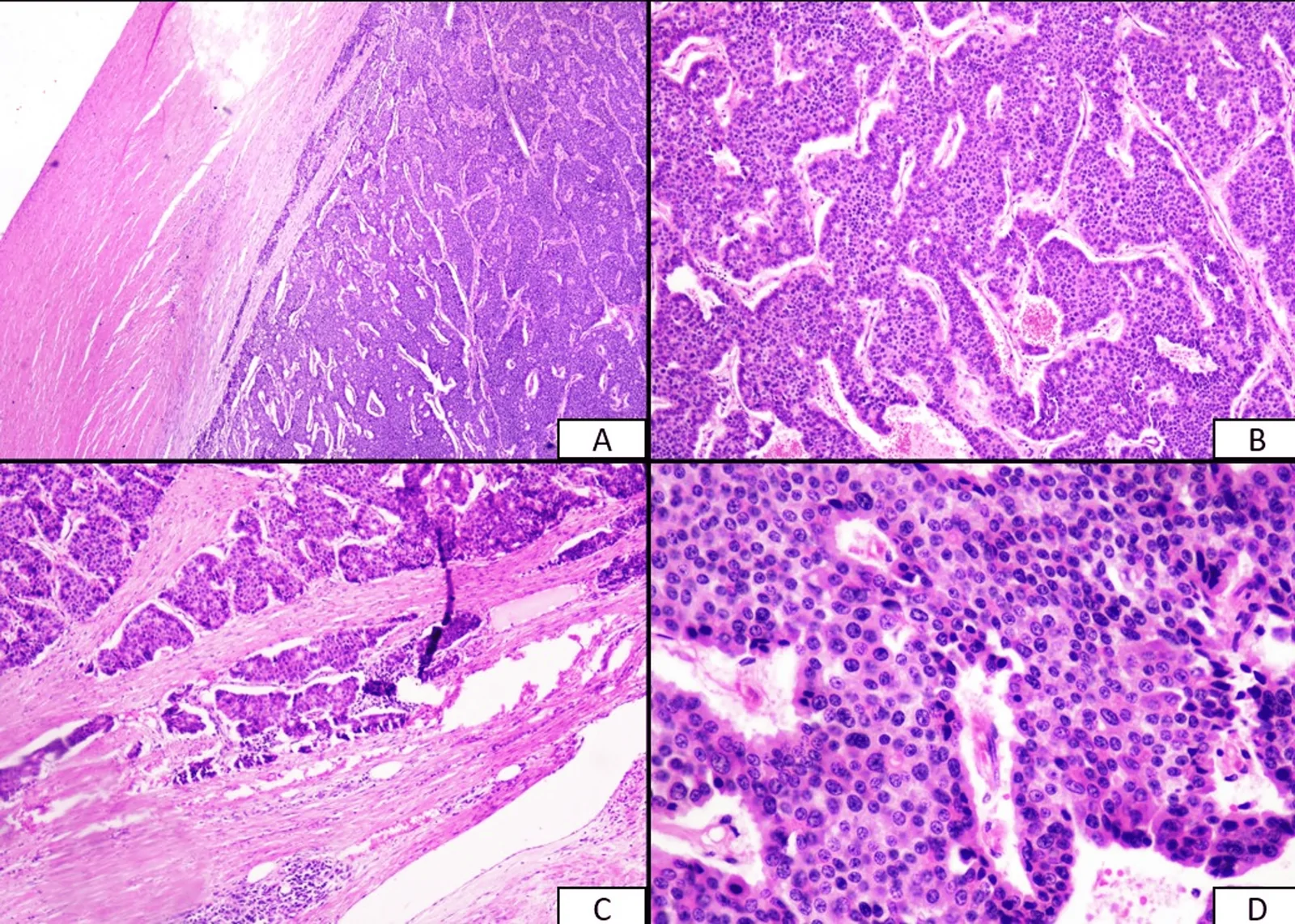
Histopathological examination (HPE) images (A, B) HPE shows a malignant neoplasm comprising neoplastic cells arranged in nests, trabeculae and rosettes, separated by collagenous stroma (A. x40, B. x100, H&E) (C) HPE shows presence of lymphovascular emboli (x100, H&E) (D) HPE shows tumor cells having round to ovoid predominantly monomorphic nuclei with focal abrupt anisonucleosis, fine stippled chromatin, inconspicuous nucleoli, 1-2 mitoses per 10 high-power fields (HPFs) and moderate amount of eosinophilic cytoplasm (x400, H&E).

**Figure 5:**
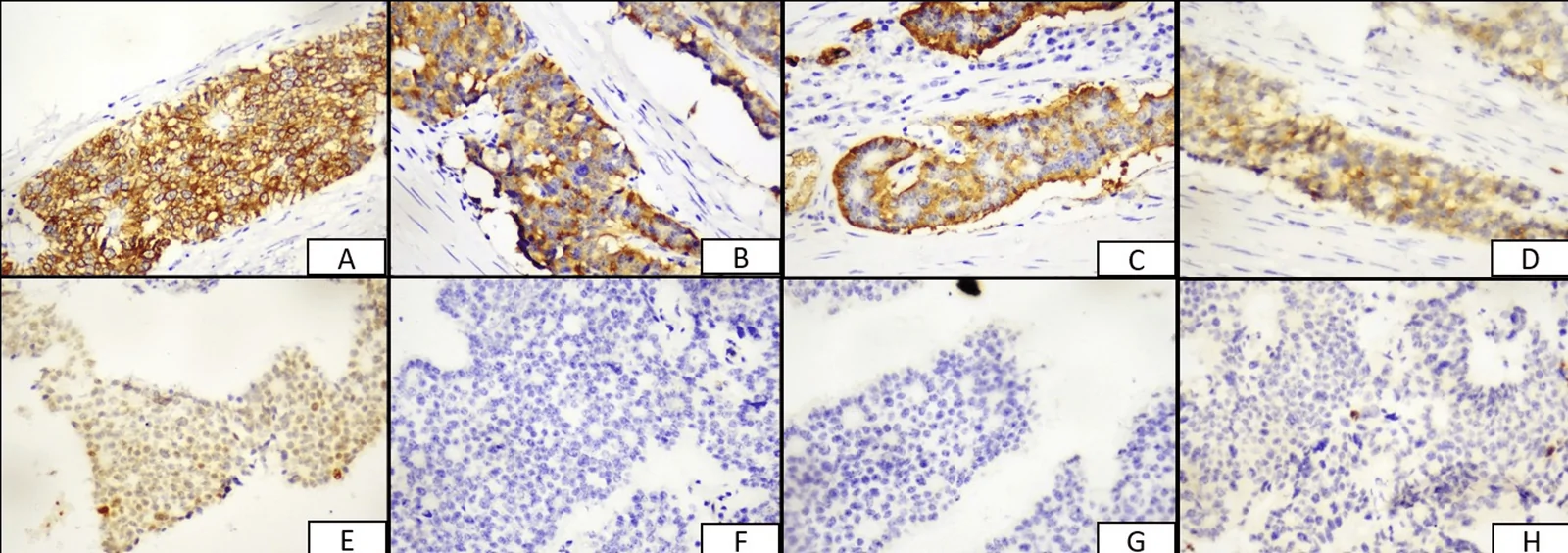
Immunohistochemistry (IHC) IHC shows tumor cells staining positive for (A) Pan-cytokeratin, (B) Synaptophysin, (C) Chromogranin, (D) CD56, and (E) INSM1, along with negative staining for (F) DOG1, (G) S100 and (H) a low Ki67 of <2% (x400, IHC).

Gross pathological examination of the excised specimen revealed a globular soft tissue mass measuring 6 × 4.5 × 3.5 cm. Microscopic examination demonstrated a WHO Grade I well-differentiated NET (G1), comprising neoplastic cells arranged in nests, trabeculae, and rosettes (Figure 4). Lymphovascular emboli were identified; however, resection margins were tumor-free. Immunohistochemistry demonstrated positivity for pan-cytokeratin, synaptophysin, chromogranin, INSM1, and CD56, with negativity for S100 and DOG-1, consistent with a diagnosis of NET (Table 1, Figure 5).

**Table 1:** Immuno-histochemical profile of mesenteric neuroendocrine tumor.

Marker	Result
Pan-cytokeratin	Positive
Synaptophysin	Positive
Chromogranin A	Positive
INSM1	Positive
CD56	Positive
S100	Negative
DOG-1	Negative
Ki67	Low

During the first two postoperative weeks, the patient remained asymptomatic. Technetium-99m somatostatin receptor scintigraphy and ⁶⁸Ga-DOTATATE positron emission tomography (PET)-CT were planned to assess for residual tumor and somatostatin receptor expression status; however, the patient was subsequently lost to follow-up.

## Discussion

Primary mesenteric NETs, defined as neuroendocrine neoplasms arising within the mesentery without a discernible gastrointestinal, pancreatic, or other primary site, are among the rarest variants of NETs [2]. The majority of mesenteric NETs encountered in clinical practice represent metastatic deposits from ileal NETs, which characteristically produce a desmoplastic reaction in the mesentery [4]. Distinguishing a true primary mesenteric NET from mesenteric metastasis of a small, occult ileal primary is therefore a significant diagnostic challenge.

In the present case, thorough intraoperative exploration, including examination of the entire length of the small and large bowel, stomach, liver, and pancreas, identified no additional lesions. The CECT findings of a stalk connecting the mass to the terminal ileum raised the possibility of an ileal primary; however, no mucosal or intramural ileal lesion was identified. The absence of a primary bowel lesion, along with the isolated mesenteric location, supports the diagnosis of a primary mesenteric NET in this case, though definitive exclusion of a small occult primary requires thorough pathological sampling.

The postoperative hypertension observed in this patient is a recognized, albeit rare, complication following resection of functioning NETs, attributed to catecholamine release, serotonin excess, or rebound vasoconstriction following removal of a vasodilatory tumor [5]. The presence of lymphovascular emboli in a WHO Grade I tumor warrants careful postoperative surveillance despite the low-grade histology.

Complete surgical resection with tumor-free margins, as achieved in this case, is the mainstay of curative treatment for localized mesenteric NETs. Postoperative functional imaging with somatostatin receptor scintigraphy or ⁶⁸Ga-DOTATATE-peptide PET-CT is essential for staging, detection of occult primaries, and surveillance for recurrence [6].

## Conclusions

Primary mesenteric NET is an exceptionally rare entity that poses significant diagnostic and therapeutic challenges. Accurate diagnosis requires a combination of cross-sectional imaging, thorough intraoperative exploration, histopathological evaluation, and immunohistochemical profiling. Complete surgical excision remains the treatment of choice, and postoperative surveillance with functional imaging is imperative to guide further management. Reporting of such rare cases contributes to the growing body of literature that may inform future diagnostic and management guidelines.
